# Simvastatin Reduces Neutrophils Infiltration Into Brain Parenchyma After Intracerebral Hemorrhage via Regulating Peripheral Neutrophils Apoptosis

**DOI:** 10.3389/fnins.2018.00977

**Published:** 2018-12-18

**Authors:** Jianbo Zhang, Xia Shi, Na Hao, Zhi Chen, Linjie Wei, Liang Tan, Yujie Chen, Hua Feng, Qianwei Chen, Gang Zhu

**Affiliations:** ^1^Department of Neurosurgery, Southwest Hospital, Third Military Medical University (Army Medical University), Chongqing, China; ^2^Department of Nutrition, Southwest Hospital, Third Military Medical University (Army Medical University), Chongqing, China; ^3^Department of Orthopedics, Chongqing Hospital of Traditional Chinese Medicine, Chongqing, China; ^4^Department of Neurosurgery, The 452 Hospital of Western Air Force, Chengdu, China

**Keywords:** intracerebral hemorrhage, statins, polymorphonuclear neutrophils, apoptosis, inflammation

## Abstract

Statins, known for their lipid-lowering effects, also have immunomodulatory properties. This study aims to examine whether systematic simvastatin administration could decrease polymorphonuclear neutrophils (PMNs) infiltration into brain tissue, as well as alleviate neuroinflammation in a rat model of intracerebral hemorrhage (ICH). The ICH model was induced in adult male Sprague–Dawley rats by an injection of autologous blood. Animals randomly received simvastatin (i.p. 2 mg/kg) or vehicle daily from 5 days before ICH until sacrificed. Routine blood counts, brain water content, neurological scoring, immunofluorescence and RT-PCR were conducted to evaluate the anti-inflammatory effect of simvastatin following ICH. Furthermore, flow cytometric and western blotting analysis were implemented for elucidating the mechanisms involved in simvastatin-induced reduction of neutrophil brain-invading. Elevated PMNs count and neutrophil-to-lymphocyte ratio in circulation were detected in rat model of ICH, which was reversed by using simvastatin. Simvastatin effectively alleviated PMNs infiltration and proinflammatory factors release in perihematomal area, as well as attenuated ICH-induced brain edema and neurological deficits. Simvastatin significantly downregulated the expression of antiapoptotic protein-Mcl-1 while increased the level of proapoptotic protein-Bax and cleaved caspase 3 in PMNs. Simvastatin treatment significantly alleviated PMNs brain-infiltrating and subsequent neuroinflammatory reaction after ICH, in part by accelerating peripheral PMNs apoptosis through disorganized the expression of apoptotic related proteins. Our data provided new evidence for simvastatin application on patients with ICH.

## Introduction

Intracerebral hemorrhage (ICH) accounts for 10–15% of all strokes, and its mortality and morbidity far exceed ischemic strokes (Qureshi et al., 2009). The lack of a specific therapeutic target in the treatment of ICH has increased the need for new treatment options (Xi et al., 2014; Joseph et al., 2016). More and more studies have provided evidence for supporting the key role of neuroinflammation in secondary brain injury following ICH (Zhou et al., 2014).

After ICH, the microglia around the hematoma takes the lead in response to injury, and then releases a variety of chemokines to recruit peripheral inflammatory cells migrate into lesion area of brain (Wan et al., 2016). Among them, the polymorphonuclear neutrophils (PMNs) first arrived perihematomal region. Both previous clinicopathological findings and recent animal experiments suggested that PMNs began to infiltrate into the brain at 6 h after ICH and reached peak on 1˜3 day (Wang, 2010). Subsequently, a large number of proinflammatory cytokines released by PMNs will activate the adjacent microglia/macrophages, leading to the release of more proinflammatory factors and resulting in the “inflammatory cascade effect,” which worse the prognosis of patients with ICH (Iadecola and Anrather, 2011; Behrouz, 2016). Taken together, PMNs is considered as the “fuse” of inflammation in the central nervous system (CNS), which could be a promising therapeutic target for ICH. Many years ago, some investigators had tried to deplete peripheral PMNs in a rat model of ICH (Moxon-Emre and Schlichter, 2011; Sansing et al., 2011). They found that PMNs specific antibody injection effectively diminished PMNs and monocyte infiltration into brain, attenuated BBB breakdown and improved functional outcome. However, to some extent, the proper dosage of PMNs antibody is hard to determine among human beings, and rapid loss of PMNs in circulation may breakdown the immune system homeostasis and lead to infection. Therefore, new therapeutic methods were still needed for suppressing PMNs brain-invading post-ICH.

In the past few years, several clinical studies have pointed out that the elevated PMNs count and neutrophil-to-lymphocyte ratio (NLR) in circulation was closely related to the poor prognosis after ICH (Lattanzi et al., 2016; Wang et al., 2016; Gusdon et al., 2017; Tao et al., 2017). However, the mechanisms responsible for this relationship remain poorly characterized. Recently, a randomized, double-blind clinical study reported that systematic administration of simvastatin, an HMG-CoA reductase inhibitor, before cardiopulmonary bypass significantly increased the apoptotic ratio of peripheral PMNs, and ameliorated post-operative inflammation (Chello et al., 2007). Taken together, we hypothesized that simvastatin could be a safe and effective candidate for attenuating PMNs brain-infiltration and subsequent inflammatory reaction following ICH, by reducing the PMNs count and NLR in circulation through regulating the apoptosis of PMNs. The present study was designed to test whether consecutive simvastatin treatment (before and after ICH) could decline the peripheral PMNs count and NLR, by which diminishing PMNs infiltration into brain in a rat model of ICH.

## Materials and Methods

### Animals and ICH Model

Two hundred and eighty-five adult male Sprague-Dawley rats (250–350 g; the Third Military Medical University) were used. All animals were housed with a 12 h light/dark cycle and water and food provided *ad libitum*. A feedback-controlled heating pad was used to maintain rats at 37.0°C during operation. The animals were kept warm at thermostats and clear respiratory secretions after operation, then return the model rats to cages until its fully awake. Animal use procedures were in compliance with the Guide for the Care and Use of Laboratory Animals and approved by the Laboratory Animal Welfare and Ethics Committee of the Third Military Medical University (SCXK-PLA-20120011). Animals were anesthetized with pentobarbital (40 mg/kg IP), and a feedback-controlled heating pad was used to maintain body temperature at 37.0°C. The rat model of ICH was established according to our previous published work (Chen et al., 2017; Jiang et al., 2017). For the model of ICH, a cranial burr hole (1 mm) was drilled, and a 29-gauge needle was inserted stereotaxically into the right caudate nucleus (coordinates: 0.2 mm anterior, 5.5 mm ventral, and 3.5 mm lateral to the bregma). Subsequently, 100 μl of autologous arterial blood was infused in 10 min using a microinfusion pump. Then, the hole was sealed using bone wax. The sham groups received only needle injection.

### Experimental Grouping

This study was divided into 3 parts. First, to examine whether simvastatin could decrease peripheral PMNs count and PMNs brain-infiltrating, ninety rats had an intracaudate injection of 100 μl of blood. The sham control received only needle injection. The animals were randomly assigned to three groups. Group 1 received simvastatin (2 mg/kg/d, i.p.) from 5 days before ICH until sacrificed, and the control group received the same volume of vehicle. Some rats (*n* = 4 per group, each time point) were sacrificed for RT-PCR analysis of TNF-α, IL-6, CCL2 and ICAM-1 at 6, 12, and 24 h after ICH. Other animals (*n* = 5 for sham group, *n* = 8 for ICH+simva. group and ICH+Veh. group each time point) were employed for blood cell count and MPO staining at 1, 3, and 7 days after ICH. In the second part, to evaluate the neuroprotective effect of simvastatin treatment on brain injury following ICH, rats were randomly divided into three groups and treated as part I. Neurological function scoring was conducted in some rats one day before ICH and 1, 3, and 7 days following ICH (*n* = 6 per group). Other animals (*n* = 6 per group, each time point) were sacrificed for brain water content measurement at 24 and 72 h after ICH. In the third part, to explore the mechanism underlying simvastatin-mediated against peripheral PMNs brain-invading after ICH, rats were randomly divided into three groups and treated as part I. Flow cytometric analysis of peripheral PMNs apoptosis (*n* = 6 per group, each time point) and lymphocytes apoptosis (*n* = 6 per group, each time point) were conducted at days 1, 3, and 7 after ICH. Then, the level of apoptotic related proteins was detected using Western blotting analysis at 24 and 72 h after ICH (*n* = 4 per group for each time point).

### Drug Administration

Simvastatin (Sigma, United States) was prepared as a 4 mg/ml stock, as previously described (Leung et al., 2003; Jin et al., 2013). Briefly, 4 mg of simvastatin was dissolved in 100 μl of ethanol and 150 μl of 0.1 N NaOH, incubated at 50°C for 2 h, then the pH adjusted to 7.0 with 0.1 M HCl, and added water to 1 ml. This simvastatin stock solution was stored at −80°C and diluted with triple volume of sterile saline immediately before use. Animals were randomized to receive intraperitoneal injection of simvastatin (2 mg/kg/d) from 5 days before ICH until sacrificed. The dose regimen of simvastatin was referred to previous studies in rat model of ICH (Karki et al., 2009; Yang et al., 2013; Chen et al., 2017).

### Routine Blood Counts

Routine blood counts were performed as previously described (Welles et al., 2009). First, animals were anesthetized with pentobarbital, then the heart was exposed and about 4 ml fresh blood samples were collected using the EDTA-anticoagulated tube. After fully shake, 200 μl blood sample was transferred to an eppendorf tube and analyzed on the bench-top analyzer (Hemavet 950, Shandong Excellent Science Instrument Co. Ltd., CHN). Before analyzer running, the quality of machine cleaning liquid, hemolysin liquid and protective agent were checked, and then use double steamed water for calibration.

### PMNs and Lymphocytes Isolation

PMNs isolation and identification were conducted as previously described (Dyugovskaya et al., 2012). The EDTA-anticoagulated whole blood from rats was collected, and it was then mixed with dextran t-500 (1% v/w) for 30 min at 37°C. The upper leukocyte-rich layer was transferred to a new tube and centrifuged at 2000 g for 20 min at 20°C. Pellets were suspended in 2 ml salt solution balanced by D-Hanks; then, they were loaded on the top of the 2 ml Histopaque1119 and Histopaque1083 density gradient carefully and centrifuged at 700 g for 30 min at 20°C. The cells were collected in the PMN-rich layer between Histopaque1083 and Histopaque1119 and were suspended in D-Hanks. The viability of the cells was determined by Trypan blue dye exclusion. The purity of isolated PMNs was detected by Wright-Giemsa staining. PMNs’ viability and purity were more than 95%. Refer to the above methods, we also isolated lymphocytes from circulating blood of ICH rat.

### Wright-Giemsa Staining

Wright-Giemsa staining was performed standard protocols (Qin et al., 2015). Briefly, the smear of peripheral blood or isolation was staining with Wright-Giemsa stains A dye 10–15 s and then staining with Wright-Giemsa stains B dye 2 min, flush slides under running water for at least 30 s after staining.

### PMNs and Lymphocytes Apoptosis Detection

The apoptotic ratio was measured by flow cytometry as previously described (Jin et al., 2013). The D-Hanks-washed cells were incubated on ice with 5 μl Annexin V-fluorescein isothiocyanate (FITC) solution and 10 μl propidium iodide (PI) solution for 15 min in dark. Then, flow cytometry (BD LSRF Ortessa, United States) was used to analyze the apoptosis.

### Brain Water Content

Animals were decapitated under deep anesthesia 24 and 72 h after autologous blood injection for brain water content measurement. Brains were removed quickly, and the frontal poles (4 mm) were cut off. The remaining brains were divided into five parts: cerebellum, ipsilateral cortex and basal ganglia, contralateral cortex and basal ganglia. Brain samples were weighed immediately to record the wet weight and were then dried at 100° for 24 h to record the dry weight. Tissue water content was calculated as (wet weight – dry weight)/wet weight. The brain water content was measured by as previously described (Williamson and Colbourne, 2017).

### Assessment of Neurological Abnormalities

Neurological dysfunction of rats was evaluated using a modified Neurological Severity Score (mNSS) method, forelimb placing test and corner test as described previously (Hua et al., 2002; Liew et al., 2012). Briefly, the assessment was performed on day 1 before and on days 1, 3, and 7 after ICH. First, the mNSS is a composite test of motor, sensory, and balance functions. Neurological function was graded on a scale of 0–18 (normal score, 0; maximal deficit score, 18). Then, each rat was tested 10 times for each forelimb, and the percentage of trials in which the rat placed the appropriate forelimb on the edge of the countertop in response to vibrissae stimulation was determined. Next, all rats were allowed to proceed into a corner, whose angle was 30°. To exit the corner, the rat could turn to either the left or the right, and that direction was recorded. The test was repeated 10 times, with at least 30 s between trials, and the percentage of right turns was calculated. Testers were highly experienced and blinded to the condition of the animal. The mean neurological score was evaluated by 2 blinded observers.

### Cell Counts

The cell counts were performed at days 1, 3, and 7 after ICH, respectively (Chen et al., 2017). Cell counts analysis was performed as previously described. For quantification of the MPO positive cells in the perihematomal area (0.2 mm anterior to bregma), consecutive slices were made, and two sections per animal (*n* = 6 per group) with 40 μm space in between were used for cell counts. Three high-power images (40× magnification) were used for cell counting. Cell counts were performed by two researchers in a blinded manner. All measurements were repeated three times, and the mean value was used.

**Table 1:** Primers used for RT-PCR.

Gene	Primers (5′ ∼ 3′)	Primer location	Product (bp)	Genbank no.
GAPDH	GACATGCCGCCTGGAGAAAC AGCCCAGGATGCCCTTTAGT	792–883	92	NM_017008.4
IL-6	ACTTCCAGCCAGTTGCCTTCTTG TGGTCTGTTGTGGGTGGTATCCTC	87–109	110	NM_012589.2
TNF-α	CACCACGCTCTTCTGTCTACTGAAC TGGGCTACGGGCTTGTCACTC	276–300	141	NM_012675.3
ICAM-1	CTGTCAAACGGGAGATGAATGG TCTGGCGGTAATAGGTGTAAATGG	1403–1421	189	NM_012967.1
CCL2	GCATCAACCCTAAGGACTTCAGC AAGGCATCACATTCCAAATCACA	391–413	155	NM_031530.1

*GAPDH, Glyceraldehyde-3-phosphate dehydrogenase; IL-6, Interleukin-6; TNF-α, Tumor Necrosis Factor-α ; CCL2, C-C motif chemokine ligand 2; ICAM-1, Intercellular Adhesion Molecule 1.*

### Real-Time PCR

The PCR was performed and analyzed, as previously described (Tan et al., 2017). For RT-PCR of TNF-α, IL-6, CCL2, and ICAM-1 gene expression analysis, rats (4/group) were sacrificed 6, 12, and 24 h by decapitation after ICH. The brains were then dissected 2 mm anteriorly and 2 mm posteriorly to the needle entry site (easily identifiable on the brain surface) and were divided into separate hemispheres along the midline. Next, perihematomal brain tissue was used for RNA extraction. Primers were designed with the Primer3 Output program (Table 1). Total RNA was extracted using TRIzol reagent (Invitrogen). A positive standard curve for each primer was obtained using a serially diluted cDNA (complementary DNA) sample mixture. Gene expression was quantified with standard samples and normalized with glyceraldehyde 3-phosphate dehydrogenase (GAPDH). The data are expressed as normalized mRNA expression. All data analyses were performed in a blinded manner by 2 observers.

### Western Blot Analysis

Western blot analysis was performed as previously described (Zou et al., 2017). Pre-extracted and washed cells were transferred to a sterile EP tube. Then, add the prepared lysis solution to the test tube by a ratio of 1 ml/10^7^ cells. Repeatedly pipetting and mixing until all the cellular proteins precipitation. Next, cells were lysed on ice for 5 min, vortexed, and repeat three times. After a centrifugation at 12,000 rpm for 10 min under 4° condition, the supernatant was collected and stored at −80°C for Western blot analysis. Antibodies were used as follows: Rabbit anti-rat Cleaved Caspase-3 (Cell Signaling Technology, United States 1:1000), Rabbit anti-rat Mcl-1 (Cell Signaling Technology, United States 1:1000), Rabbit anti-rat Bcl-2 (Cell Signaling Technology, United States 1:1000), Mice anti-rat Bax (Abcam, United States 1:500). Immunopositive bands of horseradish peroxidase–conjugated secondary antibodies were detected with an ECL system (GE Healthcare). The relative densities of the bands were analyzed using NIH ImageJ software.

### Determination of Myeloperoxidase (MPO) Activity

MPO activity has been used as an index of neutrophil infiltration in brain tissues. In this study, we used MPO activity to indicate neutrophils infiltration in the brain tissue after injection. Immunofluorescence staining of brain tissue was performed on fixed frozen sections as previously described (Feng et al., 2017). Rats were anesthetized with pentobarbital (100 mg/kg intraperitoneal) and perfused with 4% paraformaldehyde in 0.1 mol/L pH 7.4 PBS. The brains were removed and kept in 4% paraformaldehyde for 4–6 h and then immersed in 30% sucrose for 3–4 days at 4°C. The brains were embedded in an optimal cutting temperature compound (SAKURA, United States), and 18 mm thick slices were cut using a cryostat. The slices were stored at −20° before staining. Before being blocked with 10% goat serum for 1 h at room temperature, the sections were washed with PBST (PBS with 0.3% Triton X-100) for 30 min. Then, they were incubated with antibodies against MPO (Abcam, United States 1:100) at 4° overnight. The sections were kept at room temperature for 45 min and then washed with PBS. Then, the sections were incubated with an Alexa Fluor 555-conjugated goat anti-rabbit IgG (H+L) (Beyotime, China 1:300) secondary antibody at 37°C for 3 h. Finally, cell nuclei were stained with 40,6-diamidino-2-phenylindole (DAPI). The stained sections were viewed under identical conditions using a 310 or 320 objective on a confocal microscope (LSM-780; Zeiss).

### Statistical Analyses

The values in this study are presented as mean ± SD. The data in this study are given as the mean ± SD. Data were analyzed by one-way analysis of variance, followed by Scheffe’s *post hoc* test. Differences were considered statistically significant at a *P*-value of less than 0.05.

**FIGURE 1:**
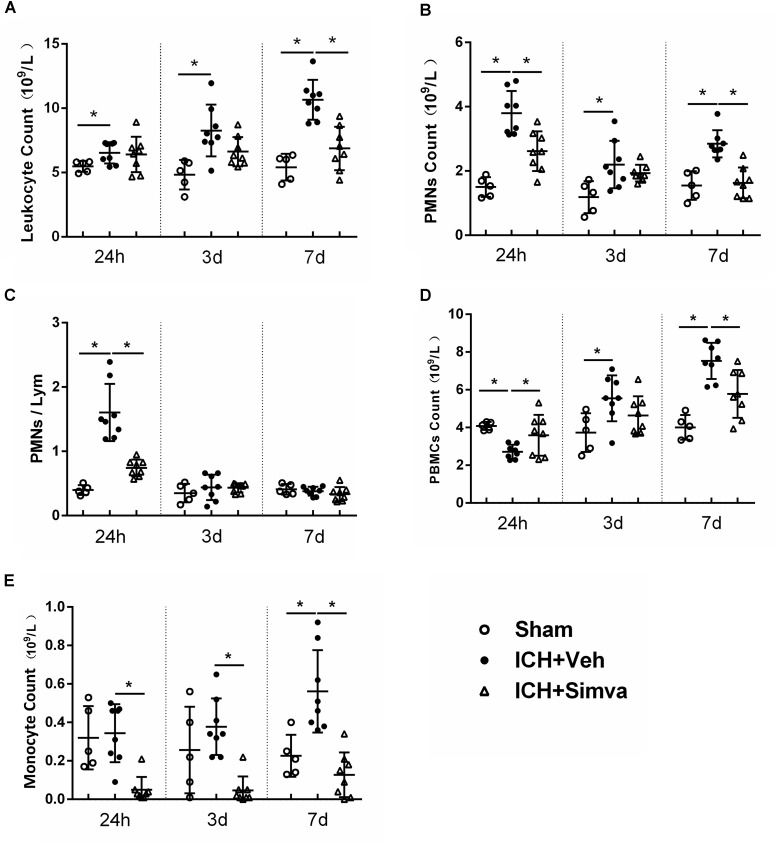
Elevated PMNs count and NLR in circulation were detected in rat model of ICH, which was reversed by using simvastatin. Analysis of peripheral leukocyte count **(A)**, PMNs count **(B)**, NLR **(C)**, PBMCs count **(D),** and monocyte count **(E)** on day1, 3, and 7 days after sham operation (*n* = 5 each time point), ICH+Vehicle (*n* = 8 each time point), and ICH+Simvastatin (*n* = 8 each time point). Values are expressed as mean ± SD; ^∗^*P* < 0.05. ICH, intracerebral hemorrhage; PMNs, polymorphonuclear neutrophils; NLR, neutrophil to lymphocyte ratio; PBMCs, peripheral blood mononuclear cells; Lym, lymphocytes.

## Results

### Elevated Peripheral PMNs Count and Neutrophil-to-Lymphocyte Ratio (NLR) Were Detected in Rat Model of ICH, Which Was Reversed by Using Simvastatin

Many clinical researches have reported that ICH patients with higher peripheral PMNs count and NLR in acute stage predicted more worse outcome (Lattanzi et al., 2016; Wang et al., 2016; Gusdon et al., 2017; Tao et al., 2017). Thus, first, we examined whether this phenomenon also exits in experimental animal model of ICH. As shown in Figure 1, compared with the sham group, the ICH rats presented higher PMNs count and higher NLR at 24 h after blood injection. Then, to test the potential effect of simvastatin on modulating leukocyte change following ICH, we conducted the dynamic blood routine analysis. The total leukocyte count in Veh-group has no difference among the Simva-, Veh- and sham- groups on day 1, but markedly increased on day 3, and reached a peak on day 7 after ICH. However, the total leukocyte count in the Simva-group remains stable on the 3 and 7 days post-ICH (Figure 1A). The PMNs count in the Simva-group was significantly lower than the control group on days 1 and 7 after ICH. Of note, 24 h after ICH, the Simva-group displayed dramatically decreased NLR compared to Vehicle controls. These results suggest that elevated PMNs count and NLR also presented in the experimental ICH model in a rat, which was effectively reversed after simvastatin treatment (Figures 1B,C). Furthermore, we also analyzed the count of peripheral blood mononuclear cells (PBMCs) and monocyte in this rat model of ICH (Figures 1D,E). PBMCs elevates on 72 h after ICH and stay high for up to 7 days. Simvastatin effectively reduced the PBMCs count on day 7 post-ICH. Notably, simvastatin significantly decreased the monocyte count from 24 h to day 7 after ICH.

**FIGURE 2:**
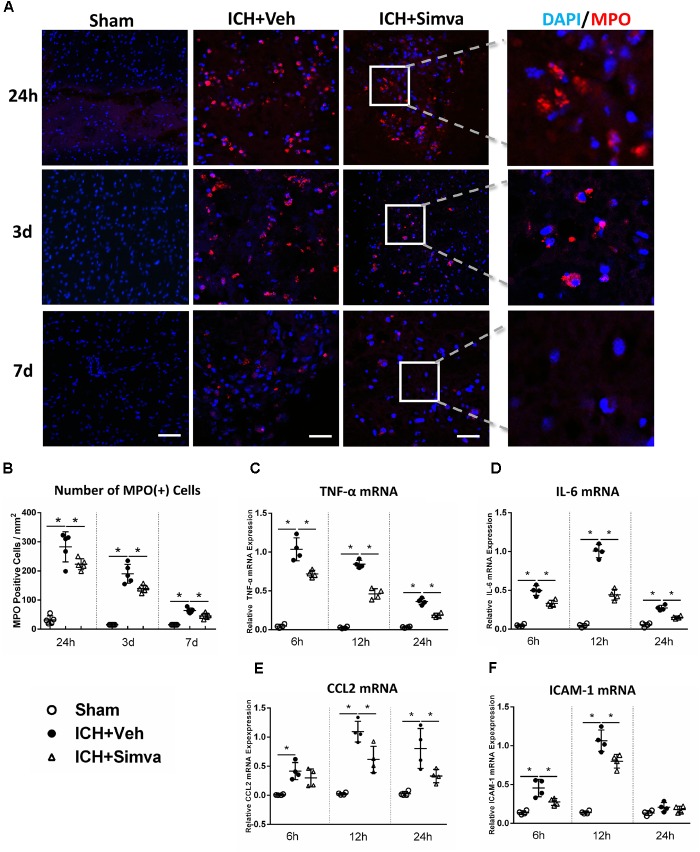
Simvastatin significantly reduced brain-infiltrating PMNs and proinflammatory mediators expression in perihematomal area after ICH. **(A)** Representative images of MPO (+) immunofluorescence around hematoma 1, 3, and 7 days after ICH. **(B)** Perihematomal MPO positive cells count at the indicated time points. Values are expressed as mean ±*SD*, *n* = 6 per group; ^∗^*P* < 0.05 versus sham group. RT-PCR analysis of TNF-α **(C)**, IL-6 **(D)**, CCL2 **(E)**, and ICAM-1 **(F)** mRNA in perihematomal tissue at 6, 12, and 24 h after ICH. Values are expressed as mean ± SD, *n* = 4 per group; ^∗^*P* < 0.05 versus sham group. The scaling bar represents 20 μm.

### Simvastatin Effectively Alleviated PMNs Brain-Infiltration and Proinflammatory Mediators Expression in Perihematomal Area

Next, we wondered whether simvastatin also changes the number of PMNs infiltration after ICH. The Laser Confocal Microscope was adopted to trace the infiltrated PMNs around hematoma on diffident time points after ICH. Compared with the Sham-group, both Simva-group and Veh-group showed a lot of MPO (+) cells in the area around the hematoma, in which the MPO (+) cells count in the Simva-group was less than the control one on the days 1, 3, and 7 post-ICH (Figures 2A,B). This data indicate that simvastatin effectively prevented peripheral PMNs infiltrating into brain parenchyma, which may be attributed by cutting down the number of PMNs in the circulation after ICH. In addition, to assess whether the degressive infiltration impacts on the proinflammatory mediators and adhesion molecule expression around the lesion, we determined the transcriptional level of some factors, like TNF-α, IL-6, CCL2, and ICAM-1 in acute stage of ICH. RT-PCR analysis showed a lower expression level of all above chemokines in the Simva-group than in the control at 6, 12, and 24 h after ICH (Figures 2C–F), suggesting simvastatin relieved the early neuroinflammatory response to ICH in some extent.

**FIGURE 3:**
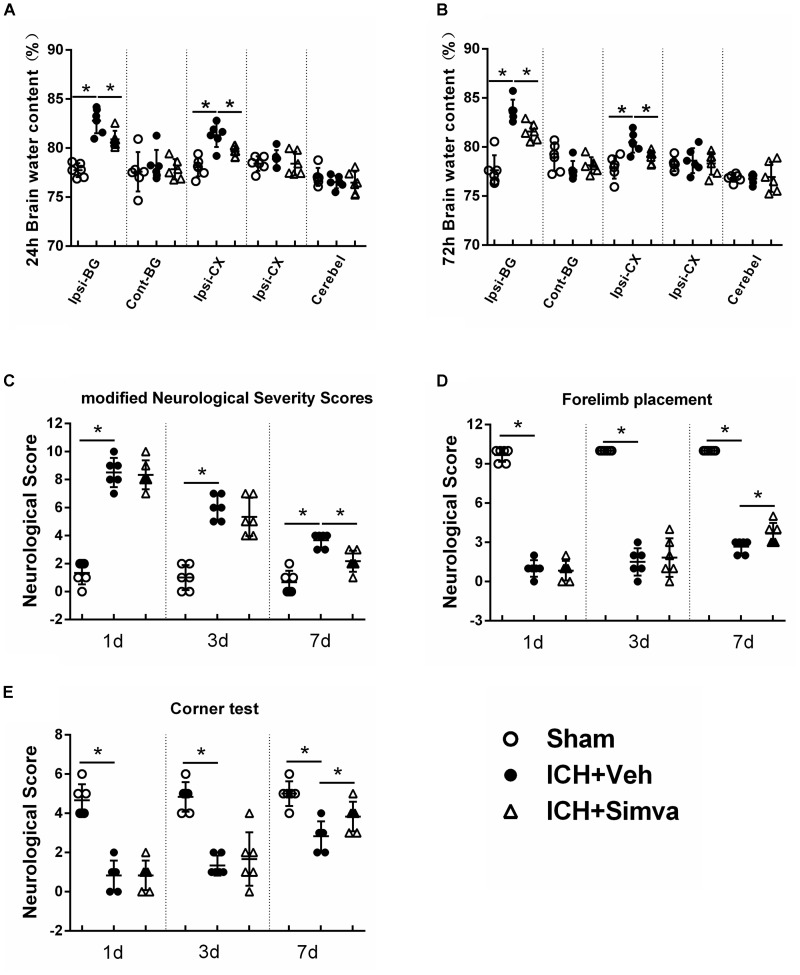
Simvastatin significantly attenuated ICH-induced brain edema and neurological deficits. Measurement of brain water content at 24 h **(A)** and 72 h **(B)** after ICH. Values are expressed as mean ± SD, *n* = 6 per group; ^∗^*P* < 0.05 versus sham group; ^#^*P* < 0.05 versus ICH-Veh group. Ipsi-BG, ipsilateral basal ganglia; Con-BG, contralateral basal ganglia; Ipsi-CX, ipsilateral cerebral cortex; Con-CX, contralateral cerebral cortex; Cerebel, cerebellum. Neurofunctional assessment by using mNSS **(C)**, Forelimb Placement Test **(D)** and Corner Test **(E)** at the indicated time points after ICH. Values are expressed as mean ± SD, *n* = 6 per group; ^∗^*P* < 0.05 ICH-Veh group versus Sham group.

### Simvastatin Significantly Attenuated ICH-Induced Brain Edema and Neurological Deficits

To further investigate the neuroprotective effect of simvastatin on ICH rats, we measured the brain water content of animal models and calculated their neurological scorings on corresponding time points post-ICH. The brain water contents in both Veh-group and Simva-group showed an obvious increase after blood injection when compared with the Sham-group. Notably, the brain water content of ipsilateral hemisphere (basal ganglia, cortex) in the Veh-group was significantly higher than the treatment group at 24 and 72 h after ICH (Figures 3A,B). As shown in Figure 3C, both Veh-group and Simva-group presented higher neurological score than the Sham-group at days 1, 3, and 7 after ICH, in which the neurological score of treatment group was significantly lower than controls at day 7. Furthermore, higher neurological scores were observed in the Simva-group than the Veh-group during the forelimb placement test (Figure 3D) and corner test (Figure 3E) at day 7 after ICH. Our above data indicate that simvastatin administration distinctly ameliorated brain edema and improved neurological function following ICH.

**FIGURE 4:**
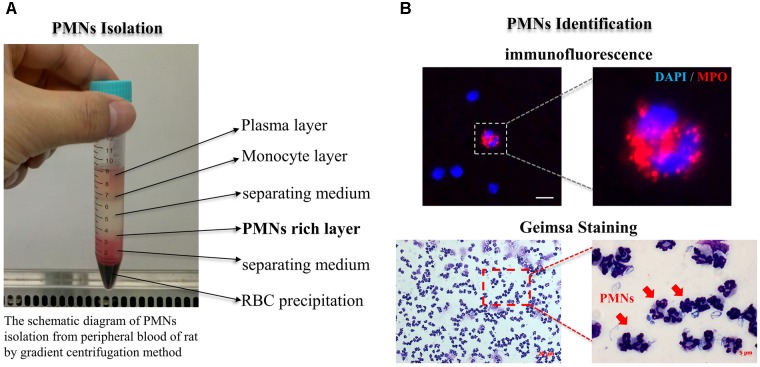
PMNs isolation and identification. **(A)** The schematic diagram of PMNs isolation from peripheral blood of rat by gradient centrifugation method; **(B)** PMNs well identified by using immunofluorescence and Giemsa staining methods. PMNs presented the feature typical for PMNs-multilobal nuclei at high magnification. The scaling bar represents 5 μm in immunofluorescence image.

### Simvastatin Broke the Balance Between Apoptotic Related Proteins, Mcl-1/Bax, Which Then Driving PMNs Apoptosis

**FIGURE 5:**
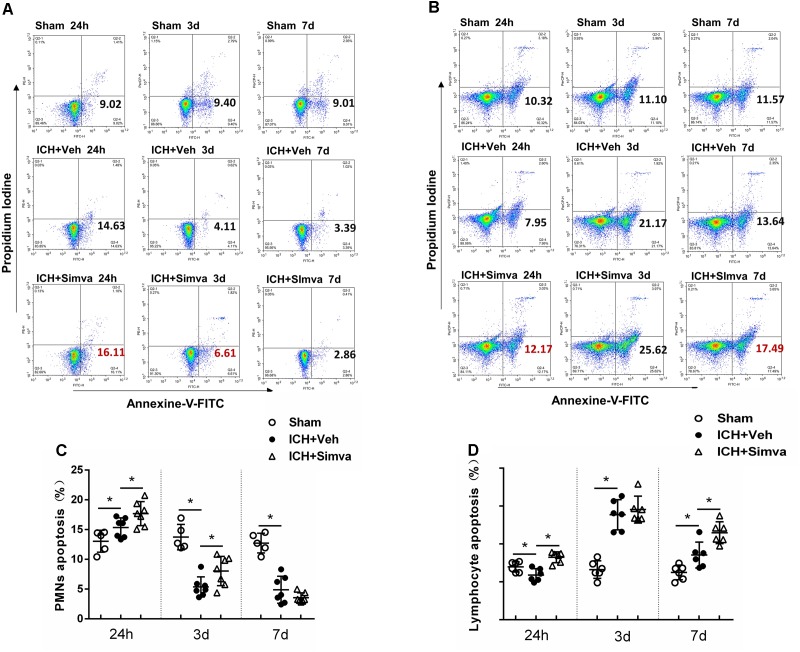
Simvastatin accelerated peripheral PMNs apoptosis after ICH, which in part by broke the balance between antiapoptotic proteins and proapoptotic proteins. Representative flow cytometric dot plots showing circulating PMNs **(A)** and lymphocytes **(B)** apoptosis, which freshly isolated from sham control rat and ICH rat treated with vehicle or simvastatin. Annexin V+ and PI- cells were considered early apoptotic cells (lower right quadrant). Flow cytometric analysis of peripheral PMNs **(C)** and lymphocytes **(D)** in the simvastatin- and vehicle-treated rat days 1, 3, and 7 after ICH. The apoptotic ratio was calculated from the ratio of apoptotic cells to total cells counted. Values are expressed as mean ± SD, *n* = 6 per group; ^∗^*P* < 0.05.

**FIGURE 6:**
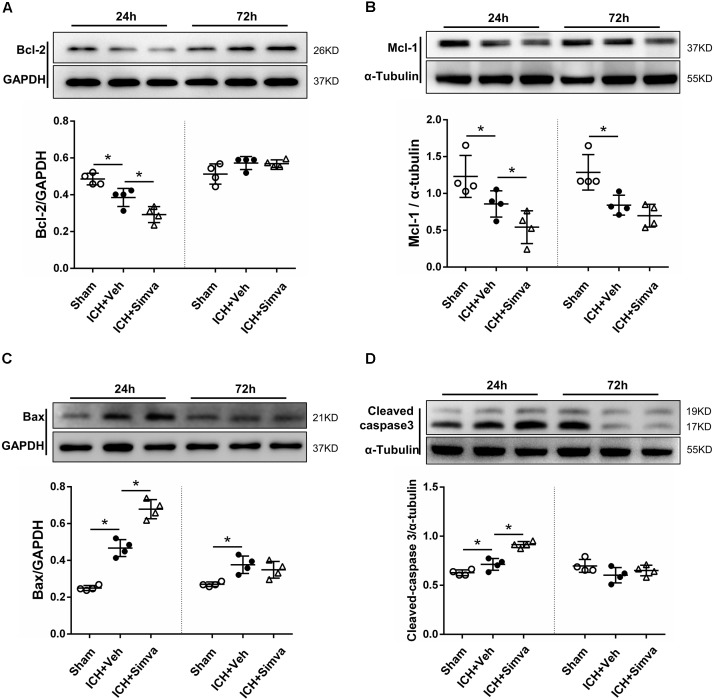
Western blot analysis of the levels of apoptotic related proteins in PMNs after ICH. Representative images and quantitative analysis of Bcl-2 protein **(A)**, Mcl-1 protein **(B)**, Bax protein **(C)**, and caspase 3 protein **(D)** in the isolated peripheral PMNs at 24 h and day 3 after ICH. Values are expressed as mean ± SD, *n* = 4 per group; ^∗^*P* < 0.05 versus sham control.

After isolated from the circulating blood of rat, PMNs were identified by using DAPI and Giemsa staining. As shown in Figure 4, at high magnification, PMNs showed the feature typical for PMNs-multilobal nuclei. Compared with the sham control, the other two experimental groups showed higher apoptotic ratio at 24 h after ICH, but obviously declined at days 3 and 7. Thereinto, the simvastatin group presented a more significantly elevated apoptotic ratio at 24 h, whereas a less ratio decreasing than the vehicle control at day 3 (Figures 5A,C). Our data indicated that simvastatin markedly enhanced the apoptosis of PMNs in the acute phase of ICH, and maintained PMNs under a higher level of apoptosis than control groups in the subacute stage, which in some extent cut down the lifespan of peripheral PMNs and shortened the time course of neuroinflammatory reaction following ICH. As shown in Figures 5B,D, simvastatin also effectively increased the apoptotic ratio of lymphocytes on days 1 and 7 post-ICH, suggesting simvastatin has potential pro-apoptotic effect on various subtypes of leukocyte not just on PMNs. Previous researches have demonstrated that the balance between antiapoptotic proteins and proapoptotic proteins is closely associated with the fate of PMNs (Dzhagalov et al., 2007; Dyugovskaya et al., 2012). Therefore, to further elucidate mechanisms for simvastatin-induced PMNs apoptosis, we traced the level of apoptotic related proteins expression in the peripheral PMNs isolated from ICH rat. As displayed in Figures 6A,B, the expression of antiapoptotic proteins, Bcl-2 and Mcl-1, was markedly inhibited at 24 h after ICH, while the proapoptotic proteins, Bax, was significantly upregulated. In comparision with control group, the ICH rats treated with simvastatin showed less antiapoptotic proteins and more proapoptotic proteins expression in peripheral PMNs. In addition, the level of cleaved caspase 3 in Simva-group was higher than the Veh-group at 24 h after ICH. Quantification of these apoptotic related proteins levels by Western blot indicated that simvastatin broke the balance between Mcl-1 and Bax on mitochondria, leading to more caspase 3 release and activation, which then initiated the apoptosis of PMNs after ICH (Figures 6C,D). In addition, we also analyzed the protein level of BCL2, MCL1, Bax and Caspase 3 expression on day 3 after ICH. Notably, as displayed in Figures 6A–D, simvastatin presented insignificant effect on the expression of apoptotic related proteins.

**FIGURE 7:**
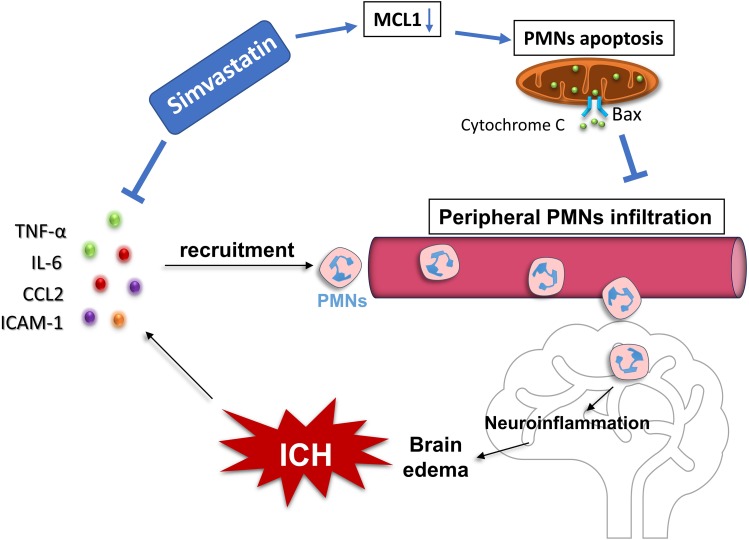
Proposed mechanism underlying simvastatin-mediated against PMNs brain-invading after ICH. It mainly depends on two approaches. On the one hand, simvastatin significantly inhibited the transcription of chemokines and vascular cell adhesion molecule, such as TNF-α, IL-6, CCL2, and ICAM-1, which subsequently reduced the chemotaxis and recruitment of peripheral neutrophil into brain after ICH. On the other hand, through down-regulated the expression of antiapoptotic protein-Mcl-1 while increased the level of proapoptotic protein-Bax expression in PMNs, resulting in more cytochrome C releasing out from mitochondria and caspase 3 activating, which then promoted the apoptosis of PMNs and cut down the number of circulating PMNs.

## Discussion

In 2016, a research team from Italy reported that in patients with acute ICH, higher PMNs, lower lymphocytes, and higher neutrophil-to-lymphocyte ratio (NLR) in peripheral circulating blood predicted worse 3 months outcome (Lattanzi et al., 2016). More Recently, a research further presented that higher NLR is independently associated with edema growth following ICH (Gusdon et al., 2017). These clinical trials suggest that peripheral PMNs count and NLR may be closely related to the prognosis of ICH. However, the mechanisms responsible for this relationship remain poorly characterized. Thus, we tested it for the first time in an experimental animal model of ICH. Notably, in this study, we observed the similar trend like in ICH patients, all ICH rats presented higher leukocyte count, PMNs count and NLR when compared with the control groups. Our data indicated that this animal model of ICH successfully reproduced the clinical situation in human beings. As PMNs plays a key role in neuroinflammation after ICH, inhibiting the migration of PMNs into brain could be a promising therapy for ICH. Furthermore, we also found that PBMCs elevates on 72 h after ICH and stay high for up to 7 days. These data partially fit with other literature reports, showing that after acute condition (miocardila infarction), PBMCs elevates within hours and stay high for up to 72 h (Nahrendorf et al., 2010). This discrepancy may be attributed to the difference between “ischemic” and “hemorrhagic” pathological nature here.

In normal conditions, PMNs lifespan was about 8–20 h in circulation. But in pathology situation, inflammatory response will delay the apoptosis of PMNs and extends its lifespan, such as arthritis, diabetes, Soehnlein et al. (2017). On this basis, we thought that is there any safe drug could accelerate PMNs apoptosis and promotes inflammation resolution after ICH. Recently, a randomized, double-blind clinical study reported that simvastatin therapy before cardiopulmonary bypass significantly increased peripheral PMNs apoptotic ratio and reduced the post-operative peak values of interleukin (IL)-6 and IL-8 (Chello et al., 2007). In this study, we observed that animals treated with simvastatin displays higher PMNs apoptotic ratio than control groups, as well as lower PMNs count and NLR in circulation. In addition, fewer PMNs invasion and lower transcriptional level of proinflammatory factors were detected in the perihematomal area after simvastatin administration. According to previous studies, CCL2 and ICAM-1 were closed with PMN invasion to lesion site after injury (Yang et al., 2005; Reichel et al., 2009). Thus, in present study, we detected the mRNA level of CCL2 and ICAM-1 in the perihematomal area at 6, 12, and 24 h after ICH. The data shows that ICH significantly upregulated the mRNA level of these chemokines and vascular adhesion molecule, which is reversed by the treatment with simvastatin. Our data indicated that simvastatin-mediated against PMN infiltration into brain may in part by inhibiting RNA transcription of CCL2 and ICAM-1 following ICH. In view of this, we proposed that simvastatin could be a safe and effective mean for attenuating PMNs brain-infiltration and subsequent inflammatory reaction following ICH.

According to previous literature, neutrophils can promote inflammatory mediators release that help recruit monocytes/macrophages, amplifying the inflammation. When neutrophils adhere to the endothelial surface, the contents of secretory granules are released, including cationic antimicrobial protein of 37 kd (CAP37)/azurocidin and proteinase 3, leading to endothelial cell activation, increased cellular adhesion molecule expression (such as ICAM-1), and increased monocyte adhesion (Lee et al., 2003). Neutrophils also contribute to monocyte chemotaxis by releasing LL-37 (De et al., 2000) and cathepsin G (Sun et al., 2004), and enhancing endothelial cell release of monocyte chemoattractant protein-1 (MCP-1) (Taekema-Roelvink et al., 2001). Consistently, in a rat model of ICH, selective depletion of neutrophils resulted in decreased infiltration of monocytes and macrophages into perihematomal area (Moxon-Emre and Schlichter, 2011; Sansing et al., 2011). In our study, neutrophils infiltrated into brain and promoted the mRNA transcription of CCL2 (also referred to as MCP-1), ICAM-1 and pro-inflammatory factors around hematoma. Notably, all of these effects of neutrophils were prevented by simvastatin. Taken together, our data in some extent suggest that neutrophils mobilization play a role in the process of leukocytes activation following ICH.

Previous researches have demonstrated that the balance between Bax/Mcl-1 has a key role in modulating PMNs apoptosis or survival (Dyugovskaya et al., 2012). PMNs constitutively express the proapoptotic members of the Bcl-2 family, including Bax, Bad, Bak, Bid, and Bik. While Mcl-1 is a member of the antiapoptotic proteins of Bcl-2 family, which plays its role by combining with Bax that expression on the outer membrane of mitochondria, forming heteromeric two dimers and stabilizing mitochondrial membrane potential. Then, preventing mitochondrial release of cytochrome C, and ultimately reducing caspase 3 activation to prevent PMN apoptosis. Recent clinical studies have found that the level of Mcl-1 expression is negatively correlated with the severity of PMNs apoptosis (Moulding et al., 1998). Much more, animal study further confirmed that conditional knockout of Mcl-1 gene significantly accelerated the apoptosis of PMN (Dzhagalov et al., 2007). Therefore, to illuminate how simvastatin promoted PMNs apoptosis, we conducted Western blotting analysis of apoptotic related proteins in present study. Our data show that the level of antiapoptotic protein, Mcl-1, was down-regulated while the expression of proapoptotic protein, Bax, increased after simvastatin used.

HMG-CoA reductase, as a rate-limiting enzyme, catalyzes the cholesterol synthesis pathway in the liver and other tissues. By inhibiting HMG-CoA reductase, statins lower cholesterol levels and might also reduce intracellular levels of isoprenoids, such as farnesyl pyrophosphate and geranylgeranyl pyrophosphate (Weitz-Schmidt, 2002). Isoprenoids are necessary for the post-translational lipid modification (prenylation) of a variety of proteins, thus anchoring them to the cell membrane (Zhang and Casey, 1996). Protein targets include the small guanosine triphosphate (GTP)-binding proteins that have a key role in signal transduction pathways that regulate cell proliferation, cell differentiation, vesicular transport and apoptosis. In addition to their cholesterol-lowering activity, statins also have pleiotropic effects, like anti-inflammation, promoting hematoma absorption, neurogenesis and neuroplasticity in some CNS diseases via the non-cholesterol pathway (Karki et al., 2009; van der Most et al., 2009; Yang et al., 2013; Wang et al., 2018). In the past few years, some retrospective clinical studies on ICH have suggested that statin use may has potential to reduce mortality, improve outcomes, with no risk of inducing rehemorrhage (Flint et al., 2014; Pan et al., 2014; Chen et al., 2015). However, to date, no randomized clinical trials have examined the exact effect. Although preclinical studies from our research team and other centers have made some progress during the past decade, it is still a long way for statin use in patients with ICH. In this study, we observed simvastatin effectively prevented PMNs infiltration, suppressed proinflammatory factors release, and attenuated brain edema following ICH through regulating PMNs apoptosis. Thus, our results may provide another evidence for future statin use in ICH patients, and more related researches were needed to understand the potential mechanism. Interestingly, a recent work reported that systemic depletion of PMNs at 24 h after ICH exacerbated neurological deficits, suggesting that PMNs infiltration into the brain also shows some beneficial function at the subacute stage post-ICH (Zhao et al., 2018). After further investigation, they proposed that PMNs-mediated protective effect in subacute stage may through the two following ways: (1) PMNs can deliver cytoprotective lactoferrin to the ICH-affected brain, neutralizing iron and blocking its toxicity (Zhao et al., 2018). (2) ICH can also transform the phenotype of PMNs that entering brain at the later stages, which then could enhance the beneficial effects like promoted blood detoxification efficacy (Zhao et al., 2017). From the above results, we can read that peripheral PMNs are like a double-edged sword for ICH- it counteracts the iron toxicity as well banes to exacerbate the secondary neuroinflammation at the different stages following ICH. Therefore, correct therapies should be chosen at correct time when targeting circulating blood PMNs after ICH, so as to maximize the natural processes of brain repair. Contrary to traditional belief, increasing evidence support that neutrophils also play a role in chronic inflammatory disorders, such as obesity and atherosclerosis (Soehnlein et al., 2017). Soehnlein et al. (2017) highlighted some therapeutic strategies to prevent neutrophil-orchestrated chronic inflammation, such as inhibition of neutrophil extracellular trap (NET)-driven inflammation, dampening neutrophil recruitment, promoting neutrophil “reverse migration” from the tissue into the bloodstream, inhibition of cyclin-dependent kinase 9 (CDK9) promotes neutrophil apoptosis.

## Conclusion

Our data revealed that the PMNs count and NLR in circulation significantly increased in experimental ICH, which in consistent with the clinical situation occurred in ICH patients. Simvastatin effectively reduced the elevated PMNs count and NLR in circulation, and significantly inhibited PMNs brain-invading and ameliorated ICH-induced brain edema and neurological deficits in rats. Moreover, simvastatin-mediated against PMNs brain-infiltration after ICH may in part by accelerating the apoptosis of peripheral PMNs as well as reducing the mRNA level of pro-inflammatory mediators (Figure 7). Our results not only provide evidence for simvastatin use on patients with ICH, but also suggest that PMNs apoptosis regulation may be a new therapeutic target for ICH.

## Ethics Statement

All institutional and national guidelines for the care and use of laboratory animals were followed.

## Author Contributions

JZ, XS, NH, LW, LT, YC, and QC contributed to the implementation of the experiment. QC, HF, ZC, and GZ contributed to the design and paper writing.

## Conflict of Interest Statement

The authors declare that the research was conducted in the absence of any commercial or financial relationships that could be construed as a potential conflict of interest.
